# Detecting *Schisandrae Chinensis Fructus* and Its Chinese Patent Medicines with a Nucleotide Signature

**DOI:** 10.3390/genes10050397

**Published:** 2019-05-22

**Authors:** Wenjun Jiang, Li Ren, Mengyue Guo, Nitin Mantri, Sha Zhao, Xiaohui Pang

**Affiliations:** 1 Key Lab of Chinese Medicine Resources Conservation, State Administration of Traditional Chinese Medicine of the People’s Republic of China, Institute of Medicinal Plant Development, Chinese Academy of Medical Sciences & Peking Union Medical College, Beijing 100193, China; wenjunjiang0927@gmail.com (W.J.); renliwangyi@hotmail.com (L.R.); guomy0908@hotmail.com (M.G.); zhaosha919@hotmail.com (S.Z.); 2 The Pangenomics Group, School of Science, RMIT University, Melbourne 3083, Australia; nitin.mantri@rmit.edu.au

**Keywords:** Wuweizi, Chinese patent medicines, identification, nucleotide signature

## Abstract

*Schisandrae Chinensis Fructus* (Wuweizi) is often adulterated with *Schisandrae Sphenantherae Fructus* (Nanwuweizi) in the herbal market. This adulteration is a threat to clinical treatment and safety. In this study, we aimed to develop a nucleotide signature for the identification of Wuweizi and its Chinese patent medicines based on the mini-DNA barcoding technique. We collected 49 samples to obtain internal transcribed spacer 2 (ITS2) sequences and developed a 26-bp nucleotide signature (5′-CGCTTTGCGACGCTCCCCTCCCTCCC-3′) on the basis of a single nucleotide polymorphism (SNP) site within the ITS2 region that is unique to Wuweizi. Then, using the nucleotide signature, we investigated 27 batches of commercial crude drug samples labeled as Wuweizi and eight batches of Chinese patent medicines containing Wuweizi. Results showed that eight commercial crude drug samples were adulterants and one of the Chinese patent medicines contained adulterants. The nucleotide signature can serve as an effective tool for identifying Wuweizi and its Chinese patent medicines and can thus be used to ensure clinical drug safety.

## 1. Introduction

**Figure 1:**
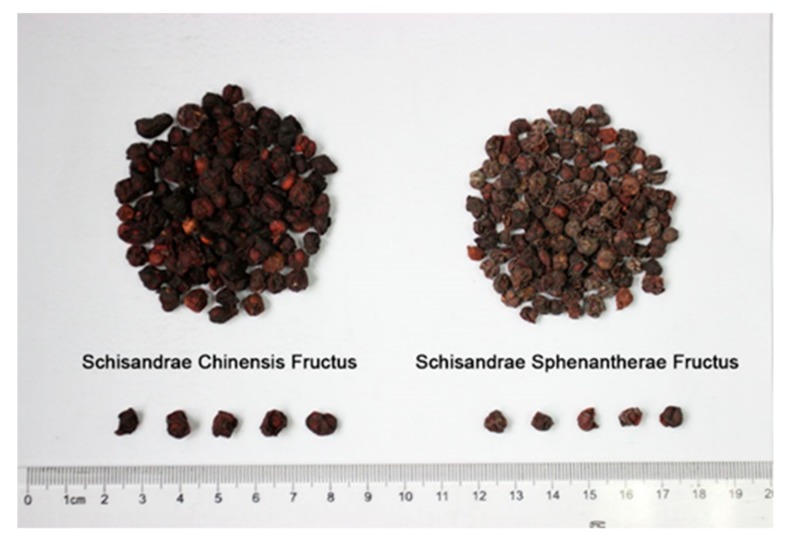
Samples of Schisandrae Chinensis Fructus and Schisandrae Sphenantherae Fructus collected from herbal market.

*Schisandra* of the family Magnoliaceae is a genus with about 30 species, two of which, namely *Schisandra chinensis* (Turcz.) Baill. and *Schisandra sphenanthera* Rehd.et Wils., have a long history of clinical use in China. *Schisandrae Chinensis Fructus*, the dry ripe fruits of *S. chinensis*, is recorded as Wuweizi in Chinese pharmacopeia. It has functions of astringing and securing, tonifying qi and engendering fluid, as well as tonifying the kidney and calming the heart [1]. As a well-known traditional Chinese medicine, it has been used for thousands of years because of its outstanding physiological as well as therapeutic effects [2]. It has also been recognized as an official medicine in the Russian Pharmacopoeia since the early 1960s [3]. Modern pharmacological research demonstrates that the fruits of *S. chinensis* contain various active components, which act as hepatoprotective [4,5], antioxidative [6], anti-inflammatory [7,8], and anti-cancer agents [9,10]. *Schisandrae Sphenantherae Fructus*, the dry ripe fruits of *S. sphenanthera*, is listed in Chinese pharmacopeia as Nanwuweizi, but has been used as Wuweizi for a long time. Nanwuweizi is inferior to Wuweizi according to the ancient pharmaceutical book Shen Nong Ben Tsao Ching and this fact has been confirmed by several clinical studies [2,11]. Nanwuweizi was listed as a new entry in Chinese pharmacopeia in 2010 because its active components, medicinal values, and qualities are different from Wuweizi [12]. However, Wuweizi is often adulterated by Nanwuweizi in the herbal market because of their similar fruit morphologies (Figure 1) and the lower price of Nanwuweizi. To guarantee their safety and efficacy, the correct identification of Wuweizi and its Chinese patent medicines is highly desired.

Distinguishing Wuweizi from Nanwuweizi through conventional taxonomic methods based on morphology is difficult because of their similar morphologies and more so when they are made into Chinese patent medicines. Previous studies reported that chemical analysis based on chromatographic techniques using schisandrin and deoxyschizandrin as markers can be used for the authentication of Wuweizi and Nanwuweizi. Such methods include thin layer chromatography, high performance liquid chromatography, and gas chromatography [13,14,15]. With the development of molecular biology techniques, polymerase chain reaction (PCR) based on molecular markers has been extensively applied in the identification of *S. chinensis* and *S. sphenanthera*. Sun et al. successfully used inter simple sequence repeat (ISSR) molecular markers to analyze population genetic variation between *S. chinensis* and *S. sphenanthera* [16]. Lee et al. demonstrated that Random amplifed polymorphic DNA (RAPD) -derived Sequence Characterized Amplified Region (SCAR) markers are useful in authenticating *S. chinensis* from other closely related species [17]. DNA barcoding has attracted considerable attention since Paul Hebert first proposed that cytochrome c oxidase 1 (CO1) can serve as a taxonomic tool for biological identification in 2003 [18]. At present, DNA barcoding has been extensively applied in the discrimination of medicinal plants and herbal materials, especially the internal transcribed spacer 2 (ITS2) barcode. Zhao et al. accurately distinguished *Acanthopanacis cortex* and its adulterants by using ITS2, indicating that ITS2 has great potential to be used for the supervision of the herbal market [19]. Guo et al. used ITS2 to authenticate medicinal plants and commercial samples in Cynanchum and showed that ITS2 has a high identification capability [20]. Li et al. successfully identified leaves of *S. chinensis* and *S. sphenanthera* using ITS2 [21].

Although DNA barcodes are effective for identifying herbs, they are difficult to be amplified from some processed samples with highly degraded DNA [22]. For instance, Lo et al. successfully amplified 88-bp DNA fragments from P. ginseng samples boiled for 120 min but failed to amplify longer fragments [23]. The reason for failure of amplifying longer fragments may be that the DNA template may have been too damaged to serve as a PCR template. Yuan et al. found that the efficiency of PCR amplification of *A. dahurica* decoction pieces decreases after baking at 80 °C [24]. The study demonstrated that short DNA sequences have advantages over long ones in terms of enhancing the success rate of amplification from processed samples. Given the difficulty the amplification of highly degraded samples poses, Meusnier et al. established a novel approach based on a DNA mini-barcode [25]. Their study indicated that the mini-barcode can be reliably obtained from archival specimens and dramatically broadens the applications of DNA barcoding. Little et al. designed a novel DNA mini-barcode and successfully applied it to the authentication of Ginkgo biloba in herbal dietary supplements [26]. Sarkinen et al. concluded that PCR success rate is strongly correlated with amplicon size; that is, the shorter fragments are more likely to achieve success [27]. The nucleotide signature, a fragment of one or more nucleotides unique to one species [28], can be used for rapidly and accurately authenticating samples with degraded DNA. Ng et al. successfully developed a nucleotide signature consisting of nine bases in the ITS region for the identification of *Aglaia stellatopilosa* [29]. Liu et al. successfully found a 27-bp nucleotide signature of American ginseng derived from the ITS2 region and used it to detect Chinese patent medicines [30]. Gao et al. successfully developed a 34-bp nucleotide signature for investigating the extracts and Chinese patent medicines containing *Lonicerae japonicae Flos* (Jinyinhua) and found many adulterated products [31]. Wang et al. successfully employed a 37-bp nucleotide signature to detect commercial products containing *Angelicae sinensis radix* (Danggui) and demonstrated that it can serve as a “species-specific marker” for Danggui [32].

To correctly identify Wuweizi and its Chinese patent medicines, the present study was designed to develop a Wuweizi-specific nucleotide signature. Finally, a 26-bp nucleotide signature (5′-CGCTTTGCGACGCTCCCCTCCCTCCC-3′), which is highly conserved and unique to *S. chinensis*, was found. To the best of our knowledge, this study is the first to use a nucleotide signature to identify Wuweizi and its Chinese patent medicines. The method based on the nucleotide signature will play a positive role in authenticating herbal materials and their products.

## 2. Materials and Methods

### 2.1. Sampling of the Materials

A total of 49 samples were collected from production areas, herbal markets, and drug stores of 14 provinces from China and Japan, including *S. chinensis* (27 fruits and one leaf sample) and *S. sphenantherae* (13 fruits, seven leaves and one seedling sample). These samples were identified by experts and voucher specimens were taken. All the corresponding voucher samples used in this study were deposited in the Herbarium of the Institute of Medicinal Plant Development, Chinese Academy of Medical Sciences, Beijing, China. ITS2 sequences were obtained and the nucleotide signature of *S. chinensis* was developed by using the above samples. The details of the reference samples for development of the nucleotide signature are listed in Table S1. A total of 143 ITS2 sequences from 18 *Schisandra* species were downloaded from GenBank and used to validate the nucleotide signature (Table S2). Then, 27 batches of commercial crude drug samples labeled as Wuweizi were gathered from ten different provinces of China and their authenticity were investigated (Table 1). Eight batches of Chinese patent medicines containing Wuweizi were purchased from Beijing and online drug stores to test if they contain adulterants (Table 2).

### 2.2. DNA Extraction, PCR Amplification, and Sequencing

#### 2.2.1. Fruit, Leaf, and Seedling

The surface of materials containing fruits and leaves were wiped with 75% ethanol for sterilization and subsequently ground into powder with FastPrepbead mill (Retsch MM400, Haan, Germany) at a frequency of 30 Hz. Genomic DNA was extracted from about 20 mg of leaf powder and 40 mg of fruit powder using the Plant Universal Genomic DNA Kit (Tiangen Biotech Co., Beijing, China) following the manufacturer’s instructions. Polymerase chain reaction (PCR) amplification and sequencing were performed according to previous research [33].

#### 2.2.2. Commercial Crude Drug Sample and Chinese Patent Medicine

**Figure 2:**
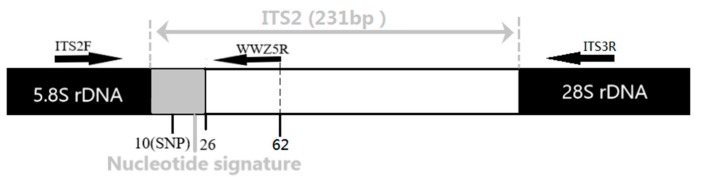
Schematic diagram showing the location of primer pairs (ITS2F/WWZ5R, ITS2F/ITS3R) and the nucleotide signature region of *S. chinensis*.

We randomly selected one sample (about 40 mg) in each batch of commercial crude drugs and 1–3 samples (200–300 mg) in each batch of Chinese patent medicines. The samples were ground into powder using a FastPrep bead mill (Retsch MM400). DNA was extracted from powder using the Plant Universal Genomic DNA Kit and amplified by ITS2F. (5′-ATGCGATACTTGGTGTGAAT-3′) /WWZ5R (5′-GCTCCTCGCAAACACCATAC-3′) primer pairs that were newly designed by Primer 6.0 software (Premier Biosoft International, Palo Alto, CA, USA) and specific for *S. chinensis* and its closely related species. The location of primer pairs is shown in Figure 2. PCR was performed in a 25 µL reaction system containing 2 µL (about 30 ng) of DNA templates, 1.0 µL of primer ITS2F/WWZ5R (2.5 µmol/L), 12.5 µL of 2× PCR Master Mix (Aidlab Biotechnologies Co., Ltd., Beijing, China), and double-distilled water. The reactions were performed with the same thermal program that was used for the other materials. Purified PCR products were sequenced in both directions with the newly designed primer pairs by the Sanger sequencing method on a 3730 XL sequencer (Applied Biosystems, Inc., Foster City, CA, USA).

### 2.3. Sequence Analysis

Raw ITS2 trace files generated from our study were assembled using CodonCode Aligner V.8.01 (CodonCode Co., Dedham, MA, USA). All the ITS2 sequences of *Schisandra* species, also including those downloaded from GenBank, were annotated as well as trimmed through a hidden Markov Model-based method to obtain the complete ITS2 regions [34]. The haplotypes of *Schisandra* species were selected by CodonCode Aligner and then aligned with MEGA 7.0 software [35]. The unique region was finally verified by BLAST analysis.

## 3. Results

### 3.1. Development of Nucleotide Signature for S. chinensis

All materials of fruits as well as leaves were successfully amplified using the primer pair ITS 2F/3R and sequenced. A total of 49 ITS2 sequences of *S. chinensis* and *S. sphenanthera* produced by experimental materials were aligned. The results of the alignment demonstrated that the intra-specific genetic divergences of *S. chinensis* and *S. sphenanthera* were low and the nucleotide (A) at position 10 was unique to *S. chinensis*. On the basis of the special SNP site, we successfully developed a 26-bp special nucleotide signature (5′-CGCTTTGCGACGCTCCCCTCCCTCCC-3′) for the identification of *S. chinensis*.

### 3.2. Evaluation of the Nucleotide Signature

**Figure 3:**
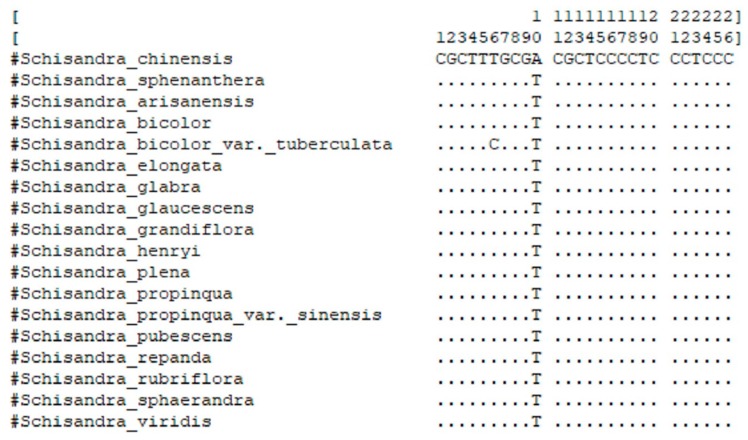
Alignment of the 26-bp nucleotide conserved region.

For the evaluation of the uniqueness and specificity of the 26-bp nucleotide signature, the 18 haplotypes of the 143 ITS2 sequences representing 18 *Schisandra* species were aligned using MEGA 7 (Figure 3) [35]. The result of the alignment confirmed the presence of one or two divergent nucleotides within the 26-bp special nucleotide signature between *S. chinensis* and other *Schisandra* species. In addition, the BLAST was conducted on GenBank using the 26-bp special nucleotide signature of *S. chinensis* as the query sequence. The results showed that the identification score of *S. chinensis* is 100%, while for other species, even for closely related species, the identification score was less than 100% (Table 3). Therefore, the 26-bp nucleotide signature, newly developed in the present study, can serve as a unique tool for distinguishing *S. chinensis* from the other species presented in GenBank.

### 3.3. Identification of Commercial Samples

#### 3.3.1. Commercial Crude Drug Samples

A total of 27 batches of commercial crude drugs labeled as Wuweizi were purchased from drug stores, herbal markets, and online stores. All the tested samples of commercial crude drugs were successfully amplified (170 bp) with the primer pair ITS2F/WWZ5R and then bidirectionally sequenced. Of the 27 samples tested, 19 were authenticated as Wuweizi and eight were adulterants. The identification results and other data of the samples investigated in our study are provided in Table 1.

#### 3.3.2. Chinese Patent Medicines

**Figure 4:**
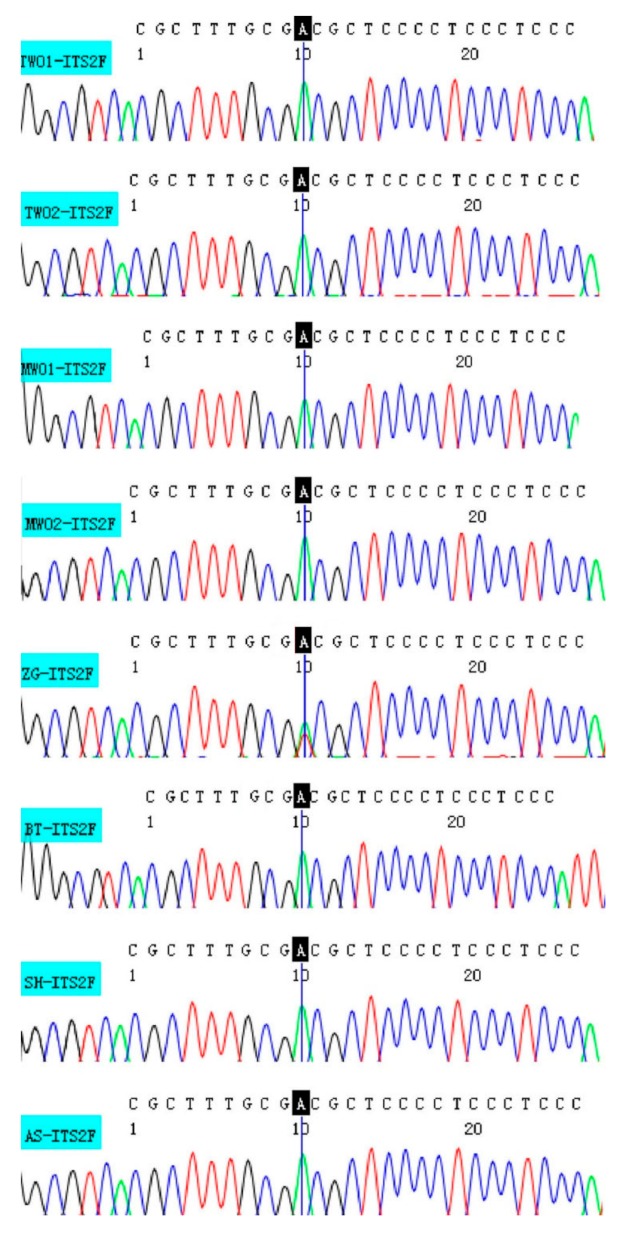
Single nucleotide polymorphisms (SNP) peaks of four batches of Chinese patent medicine containing Wuweizi.

Eight batches of Chinese patent medicines containing Wuweizi were tested by the primer pair ITS2F/WWZ5R, including Tianwang Buxin Pills, Zengguang Tablets, Maiwei Dihuang Pills, Shihu Yeguang Pills, Bantu Pills, and Anshen Capsules. The details of the Chinese patent medicines and identification results are provided in Table 2. The targeted nucleotide signature of *S. chinensis* was successfully amplified (170 bp) and sequenced from these batches. After analyzing the nucleotide signature and double peaks at the SNP position (Figure 4), we found that one batch (Zengguang Tablets) contained adulterants and seven were authentic.

## 4. Discussion

### 4.1. The Necessity of Developing Nucleotide Signature for Chinese Patent Medicines

*S. chinensis* is widely distributed in several provinces in northeastern China, eastern Russia, Japan, and Korea [36]. This species is not only a medicinal material recorded in Chinese Pharmacopeia and Russian Pharmacopoeia, but also an important raw material for various Chinese patent medicines, such as Tianwang Buxin Pills, Zengguang Tablets and Anshen Capsules. The contents of schisandrin and schisantherin A are used as the quality control indicators for Wuweizi and Nanwuweizi, respectively, in the Chinese Pharmacopoeia (2015 edition) [1]. However, Chinese patent medicines are composed of a considerable number of herbs and each one may contain dozens or even hundreds of chemical ingredients. Adulterants may occur considering the similar chemical constituents in herbal medicines from the same genus, which is a threat to drug safety and the health of consumers. Thus, it is not sufficient to identify the composition of Chinese patent medicines by content determination alone. Developing a novel approach for differentiating ingredients listed on the label is therefore highly desirable and is an initial step for ensuring the efficacy of Chinese patent medicines.

In previous research, Li et al. used ITS2 to distinguish *S. chinensis* from *S. sphenanthera* and found that ITS2 is useful for the authentication of *S. chinensis* [21]. All the materials they used were leaves, so it is not known whether ITS2 is suitable for identifying other materials of *S. chinensis*, such as herbal materials and Chinese patent medicines. Considering that the mini-DNA barcoding technique is more suitable for the identification of processed samples with heavy DNA degradation, it was used in our study. We found a 26-bp nucleotide signature unique to *S. chinensis* for the first time, which can be used to identify *S. chinensis*, including plants, crude drugs, and Chinese patent medicines.

### 4.2. Nucleotide Signature Can Effectively Detect Wuweizi and Its Chinese Patent Medicines

A total of 27 batches of commercial crude drug samples and eight batches of Chinese patent medicines were surveyed in this study. The nucleotide signature was used in the authentication of the tested samples and it was confirmed to be efficient in identifying Wuweizi. First, the newly developed nucleotide signature was verified by BLAST analysis, which displayed perfect identification capability with 100% identification efficiency at the species level. Second, presence of adulterants in the Chinese patent medicines was determined through SNP method. Chen et al. found that a mixed powder of American ginseng and Asian ginseng can be clearly detected by analyzing the double peaks at the SNP site even when the mixing ratio was as low as 5% [37]. Similarly, in our study, *S. chinensis* and other species was efficiently distinguished by analyzing the double peaks at the SNP site.

### 4.3. Nucleotide Signature Is a Powerful Technique for Herbal Market Supervision

The DNA barcoding technique has been used for investigating the commercial products in the herbal market recently. For instance, Ruhsam et al. used DNA barcoding to investigate *Eleutherococcus* and *Rhodiola* herbal supplements and found that the phenomenon of substitution or adulteration is common [38]. Han et al. utilized DNA barcoding to survey 295 herbal materials and found that approximately 4.2% of the samples are adulterants [39]. The studies revealed that DNA barcodes are useful in identifying some commercial herbs. However, it is difficult to successfully amplify DNA barcodes for Chinese patent medicines with severely degraded DNA. The nucleotide signature is a species-specific marker much shorter than DNA barcodes, which gives it an advantage in detecting the ingredients of Chinese patent medicines. In our study, the newly identified 26-bp nucleotide signature was successfully used for the authentication of Wuweizi and its Chinese patent medicines. As shown in Table 1, eight of 27 of the tested commercial crude samples were adulterants. Meanwhile, one of the eight batches of Chinese patent medicines contained adulterants. Thus, the nucleotide signature is an efficient tool for identifying samples with heavy DNA degradation, providing a useful tool for herbal market supervision.

## 5. Conclusions

Our study revealed that the adulteration of Wuweizi is not uncommon in the herbal market, emphasizing the need for market supervision. The 26-bp nucleotide signature we developed in this study can serve as a unique tool for identifying and detecting Wuweizi and its Chinese patent medicines. The nucleotide signature has potential to be used to supervise the herbal market, thereby ensuring the efficacy and safety of drugs.

## Figures and Tables

**Table 1:** Characteristics and identification results of commercial crude drug samples labeled as *Schisandrae Chinensis Fructus*.

Voucher No.	Collection Location	Genuine
DL01	Dalian, Liaoning	Yes
DL02	Dalian, Liaoning	Yes
BJ01	Beijing	No
BJ02	Beijing	Yes
XA01	Xian, Shanxi	Yes
XA02	Xian, Shanxi	Yes
WN01	Weinan, Shanxi	No
NJ01	Nanjing, Jiangsu	No
CZ01	Changzhi, Shanxi	Yes
CZ02	Changzhi, Shanxi	No
CZ03	Changzhi, Shanxi	Yes
CZ04	Changzhi, Shanxi	No
CZ05	Changzhi, Shanxi	No
CZ06	Changzhi, Shanxi	No
CZ07	Changzhi, Shanxi	No
OL01	online store (Aomiao)	Yes
OL02	online store (Caobenyunnan)	Yes
CD01	Chengdu, Sichuan	Yes
CD02	Chengdu, Sichuan	Yes
BZ01	Bozhou, Anhui	Yes
BZ02	Bozhou, Anhui	Yes
BZ03	Bozhou, Anhui	Yes
BZ04	Bozhou, Anhui	Yes
AG01	Anguo, Hebei	Yes
TH01	Tonghua, Jilin	Yes
TH02	Tonghua, Jilin	Yes
LC01	Lincun, Jilin	Yes

**Table 2:** Characteristics of eight Chinese patent medicines.

Voucher No.	Samples	Listed Ingredients on Label	Collection Location	Genuine
TW01	Tianwang Buxin Pills	*Schisandrae Chinensis Fructus*, *Salviae Miltiorrhizae Radix Et Rhizoma*, *Angelicae Sinensis Radix*, *Acori Tatarinowii Rhizoma*, *Codonopsis Radix*, *Poria, Ophiopogonis Radix*, *Asparagi Radix*, *Rehmanniae Radix*, *Scrophulariae Radix*, *Polygalae Radix*, *Ziziphi Spinosae Semen*, *Platycladi Semen*, *Platycodonis Radix*, *Glycyrrhizae Radix Et Rhizoma*, *Cinnabaris*	Beijing store	Yes
TW02	Tianwang Buxin Pills	*Schisandrae Chinensis Fructus*, *Salviae Miltiorrhizae Radix Et Rhizoma*, *Angelicae Sinensis Radix*, *Acori Tatarinowii Rhizoma*, *Codonopsis Radix*, *Poria*, *Ophiopogonis Radix*, *Asparagi Radix*, *Rehmanniae Radix*, *Scrophulariae Radix*, *Polygalae Radix*, *Ziziphi Spinosae Semen, Platycladi Semen, Platycodonis Radix*, *Glycyrrhizae Radix Et Rhizoma*, *Cinnabaris*	online shop	Yes
MW01	Maiwei Dihuang Pills	*Schisandrae Chinensis Fructus*, *Ophiopogonis Radix*, *Rehmanniae Radix Praeparata*, *Corni fructus*, *Moutan Cortex*, *Dioscoreae Rhizoma*, *Poria*, *Alismatis Rhizoma*	Beijing store	Yes
MW02	Maiwei Dihuang Pills	*Schisandrae Chinensis Fructus*, *Ophiopogonis Radix*, *Rehmanniae Radix Praeparata*, *Corni fructus*, *Moutan Cortex*, *Dioscoreae Rhizoma*, *Poria*, *Alismatis Rhizoma*	online shop	Yes
ZG	Zengguang Tablets	*Schisandrae Chinensis Fructus*, *Codonopsis Radix*, *Acori Tatarinowii Rhizoma*, *Poria*, *Alismatis Rhizoma*, *Ophiopogonis Radix*, *Lycii Fructus*, *Angelicae Sinensis Radix*, *Moutan Cortex*, *Polygalae Radix*	online shop	No
BT	Bantu Pills	*Schisandrae Chinensis Fructus*, *Rehmanniae Radix*, *Rehmanniae Radix Praeparata*, *Polygoni Multiflori Radix Praeparata*, *Angelicae Sinensis Radix*, *Salviae Miltiorrhizae Radix Et Rhizoma*, *Paeoniae Radix Alba*, *Notopterygii Rhizoma Et Radix*, *Chaenomelis Fructus*	online shop	Yes
SH	Shihu Yeguang Pills	*Schisandrae Chinensis Fructus*, *Dendrobii Caulis*, *Ginseng Radix Et Rhizoma*, *Dioscoreae Rhizoma*, *Poria*, *Glycyrrhizae Radix Et Rhizoma*, *Cistanches Herba*, *Lycii Fructus*, *Cuscutae Semen*, *Rehmanniae Radix*, *Rehmanniae Radix Praeparata*, *Asparagi Radix*, *Ophiopogonis Radix*, *Armeniacae Semen Amarum*, *Saposhnikoviae Radix*, *Chuanxiong Rhizoma*, *Aurantii Fructus*, *Coptidis Rhizoma*, *Achyranthis Bidentatae Radix*, *Chrysanthemi Flos*, *Tribuli Fructus*, *Celosiae Semen*, *Cassiae Semen*, *Bubali Cornu*, *Saigae Tataricae Cornu*	online shop	Yes
AS	Anshen Capsules	*Schisandrae Chinensis Fructus*, *Ziziphispinosaesemen*, *Chuanxiong Rhizoma*, *Anemarrhenae Rhizoma*, *Ophiopogonis Radix*, *Polygoni Multiflori Radix Praeparata*, *Salviae Miltiorrhizae Radix Et Rhizoma*, *Poria*	online shop	Yes

**Table 3:** BLAST analysis of the 26-bp unique region.

Source Species of Nucleotide Signature	Blast Result in NCBI	Number of the Species	Max Score	Total Score	Query Cover	E Value	Per. Ident
*Schisandra chinensis*	*Schisandra chinensis*	39	52.0	52.0	100%	0.0003	100%
	*Schisandra sphenanthera*	1	44.1	44.1	100%	0.065	96.15%
	*Kadsura heteroclite*	1	44.1	44.1	100%	0.065	96.15%
	*Kadsura interior*	13	44.1	44.1	100%	0.065	96.15%
	*Schisandra sphaerandra*	2	44.1	44.1	100%	0.065	96.15%
	*Schisandra henryi*	2	44.1	44.1	100%	0.065	96.15%
	*Schisandra rubriflora*	4	44.1	44.1	100%	0.065	96.15%
	*Schisandra propinqua*	2	44.1	44.1	100%	0.065	96.15%
	*Schisandra henryi*	3	44.1	44.1	100%	0.065	96.15%
	*Kadsura longipedunculata*	27	44.1	44.1	100%	0.065	96.15%
	*Kadsura heteroclita*	6	44.1	44.1	100%	0.065	96.15%

## Supplementary Materials

The following are available online at https://www.mdpi.com/2073-4425/10/5/397/s1, Table S1. Location of collection of reference samples for the development of the nucleotide signature. Table S2. Characteristics of the *Schisandra* genus derived from GenBank. Figure S1. Alignment of the ITS2 region of experimental materials.
