# Supplementary material for: Detecting Schisandrae Chinensis Fructus and Its Chinese Patent Medicines with a Nucleotide Signature
Source: Genes (Basel). 2019 May 22;10(5):397. doi: 10.3390/genes10050397 (PMC6562420; doi:10.3390/genes10050397)
Supplement: Supplementary file 1 [file genes-10-00397-s001.pdf]

Table S1. Location of collection of reference samples for the development of the nucleotide signature.

Voucher No.	Latain Name	Sampling part	Collection Set
WWZ01	*Schisandra chinensis*	fruit	Sichuan Hehuachi Herb Market
WWZ02	*Schisandra chinensis*	fruit	Sichuan Hehuachi Herb Market
WWZ03	*Schisandra chinensis*	fruit	Chengdu, Sichuan
WWZ04	*Schisandra chinensis*	fruit	Hebei Anguo Herb Market
WWZ05	*Schisandra chinensis*	fruit	Hebei Anguo Herb Market
WWZ06	*Schisandra chinensis*	fruit	Anhui Bozhou Herb Market
WWZ07	*Schisandra chinensis*	fruit	Anhui Bozhou Herb Market
WWZ08	*Schisandra chinensis*	fruit	Anhui Bozhou Herb Market
WWZ09	*Schisandra chinensis*	fruit	Anhui Bozhou Herb Market
WWZ10	*Schisandra chinensis*	fruit	Anhui Bozhou Herb Market
WWZ11	*Schisandra chinensis*	fruit	Anhui Bozhou Herb Market
WWZ12	*Schisandra chinensis*	fruit	Anhui Bozhou Herb Market
WWZ13	*Schisandra chinensis*	fruit	Anhui Bozhou Herb Market
WWZ14	*Schisandra chinensis*	fruit	Fushun, Liaoning
WWZ15	*Schisandra chinensis*	fruit	Fushun, Liaoning
WWZ16	*Schisandra chinensis*	fruit	Fushun, Liaoning
WWZ17	*Schisandra chinensis*	fruit	Yulin, Guangxi
WWZ18	*Schisandra chinensis*	fruit	Jiagedaqi, Heilongjiang
WWZ19	*Schisandra chinensis*	fruit	Yanji, Jilin
WWZ20	*Schisandra chinensis*	fruit	Changchun, Jilin
WWZ21	*Schisandra chinensis*	fruit	Changchun, Jilin
WWZ22	*Schisandra chinensis*	fruit	Changchun, Jilin
WWZ23	*Schisandra chinensis*	fruit	Changchun, Jilin
WWZ24	*Schisandra chinensis*	fruit	Changchun, Jilin
WWZ25	*Schisandra chinensis*	fruit	Changchun, Jilin
WWZ26	*Schisandra chinensis*	fruit	Dongjing, Japan
WWZ27	*Schisandra chinensis*	fruit	Shenzhen Institute For Drug Control
WWZ28	*Schisandra chinensis*	leaf	Donglingshan, Beijing
NWWZ01	*Schisandra sphenanthera*	fruit	Chengdu, Sichuan
NWWZ02	*Schisandra sphenanthera*	fruit	Sichuan Hehuachi Herb Market
NWWZ03	*Schisandra sphenanthera*	fruit	Lvye Company
NWWZ04	*Schisandra sphenanthera*	fruit	Xian, Shanxi
NWWZ05	*Schisandra sphenanthera*	fruit	Jiujiang, Jiangxi
NWWZ06	*Schisandra sphenanthera*	fruit	Anhui Bozhou Herb Market
NWWZ07	*Schisandra sphenanthera*	fruit	Anhui Bozhou Herb Market
NWWZ08	*Schisandra sphenanthera*	fruit	Anhui Bozhou Herb Market
NWWZ09	*Schisandra sphenanthera*	fruit	Shenzhen Institute For Drug Control
NWWZ10	*Schisandra sphenanthera*	fruit	National Institutes for Food and Drug Contral

NWWZ11	*Schisandra sphenanthera*	fruit	National Institutes for Food and Drug Contral
NWWZ12	*Schisandra sphenanthera*	fruit	Changzhi,Shanxi
NWWZ13	*Schisandra sphenanthera*	fruit	Changzhi,Shanxi
NWWZ14	*Schisandra sphenanthera*	leaf	Nanyang, Henan
NWWZ15	*Schisandra sphenanthera*	leaf	Lushan, Jiangxi
NWWZ16	*Schisandra sphenanthera*	leaf	Lushan, Jiangxi
NWWZ17	*Schisandra sphenanthera*	leaf	Lushan, Jiangxi
NWWZ18	*Schisandra sphenanthera*	leaf	Lushan, Jiangxi
NWWZ19	*Schisandra sphenanthera*	leaf	Shennongjia Scenic Area, Hubei
NWWZ20	*Schisandra sphenanthera*	leaf	Shennongjia Scenic Area, Hubei
NWWZ21	*Schisandra sphenanthera*	seedling	Ruili, Yunnan

Table S2. Characteristics of the *Schisandra* genus derived from GenBank.

**Voucher No.**	**Latain Name**	**GenBank Accession No.**
1	*Schisandra arisanensis*	KP689642
2	*Schisandra arisanensis*	KP689641
3	*Schisandra arisanensis*	KP689640
4	*Schisandra arisanensis*	KP689639
5	*Schisandra arisanensis*	KP689638
6	*Schisandra bicolor*	DQ342255
7	*Schisandra bicolor*	KP689682
8	*Schisandra bicolor*	KP689681
9	*Schisandra bicolor var. tuberculata*	AF263442
10	*Schisandra elongata*	KP689651
11	*Schisandra elongata*	KP689650
12	*Schisandra glabra*	DQ342254
13	*Schisandra glabra*	EF138799
14	*Schisandra glabra*	AF163720
15	*Schisandra glaucescens*	AF263439
16	*Schisandra glaucescens*	KP689649
17	*Schisandra glaucescens*	JQ712976
18	*Schisandra glaucescens*	AF163707
19	*Schisandra grandiflora*	KP689674
20	*Schisandra grandiflora*	KP689673
21	*Schisandra grandiflora*	KP689672
22	*Schisandra grandiflora*	KP689669
23	*Schisandra grandiflora*	KP689668
24	*Schisandra grandiflora*	KP689664
25	*Schisandra grandiflora*	AF163707
26	*Schisandra henryi*	KY523601
27	*Schisandra henryi*	MH270476
28	*Schisandra henryi*	MH270475

29	*Schisandra henryi*	KY884781
30	*Schisandra henryi*	KY884780
31	*Schisandra henryi*	KY884779
32	*Schisandra henryi*	KY223658
33	*Schisandra henryi*	KP689652
34	*Schisandra henryi*	KP689653
35	*Schisandra henryi*	KP689654
36	*Schisandra henryi*	KP689655
37	*Schisandra henryi*	KP689656
38	*Schisandra henryi*	AF263435
39	*Schisandra henryi*	AF163708
40	*Schisandra plena*	AF263443
41	*Schisandra plena*	KP689688
42	*Schisandra propinqua*	KP689687
43	*Schisandra propinqua*	KP689686
44	*Schisandra propinqua*	KP689685
45	*Schisandra propinqua*	KP689684
46	*Schisandra propinqua*	KP689683
47	*Schisandra propinqua*	JF978531
48	*Schisandra propinqua*	JF978530
49	*Schisandra propinqua*	JF978529
50	*Schisandra propinqua*	AF163717
51	*Schisandra propinqua*	KY884783
52	*Schisandra propinqua*	KY884782
53	*Schisandra propinqua var. sinensis*	AF263444
54	*Schisandra pubescens*	AF263436
55	*Schisandra pubescens*	KP689661
56	*Schisandra pubescens*	KP689660
57	*Schisandra pubescens*	KP689659
58	*Schisandra pubescens*	KP689658
59	*Schisandra pubescens*	KP689657
60	*Schisandra pubescens*	JF978533
61	*Schisandra pubescens*	JF978532
62	*Schisandra pubescens*	KY563602
63	*Schisandra pubescens*	AF163709
64	*Schisandra repanda*	KX815928
65	*Schisandra repanda*	KP689680
66	*Schisandra rubriflora*	AF263440
67	*Schisandra rubriflora*	KP689671
68	*Schisandra rubriflora*	KP689670
69	*Schisandra rubriflora*	KP689667
70	*Schisandra rubriflora*	KP689666
71	*Schisandra rubriflora*	KP689665
72	*Schisandra rubriflora*	KP689663

73	*Schisandra rubriflora*	KP689662
74	*Schisandra rubriflora*	KY884784
75	*Schisandra rubriflora*	KY884785
76	*Schisandra rubriflora*	KY884786
77	*Schisandra rubriflora*	KY884787
78	*Schisandra rubriflora*	KY523603
79	*Schisandra rubriflora*	KY523604
80	*Schisandra rubriflora*	AF163706
81	*Schisandra sphaerandra*	KY523607
82	*Schisandra sphaerandra*	KY523608
83	*Schisandra viridis*	AF163703
84	*Schisandra viridis*	JF978539
85	*Schisandra viridis*	JF978540
86	*Schisandra viridis*	AF263438
87	*Schisandra viridis*	KP689643
88	*Schisandra viridis*	JF978540
89	*Schisandra viridis*	JF978539
90	*Schisandra viridis*	AF163703
91	*Schisandra chinensis*	AB558158
92	*Schisandra chinensis*	KP893160
93	*Schisandra chinensis*	KP893161
94	*Schisandra chinensis*	KP893162
95	*Schisandra chinensis*	KP893163
96	*Schisandra chinensis*	KP893164
97	*Schisandra chinensis*	KP893165
98	*Schisandra chinensis*	KP893166
99	*Schisandra chinensis*	KP893167
100	*Schisandra chinensis*	KP893159
101	*Schisandra chinensis*	KP893158
102	*Schisandra chinensis*	AF163710
103	*Schisandra chinensis*	JF970271
104	*Schisandra chinensis*	MF096273
105	*Schisandra chinensis*	MF096274
106	*Schisandra chinensis*	MF096275
107	*Schisandra chinensis*	MF096276
108	*Schisandra chinensis*	MF096277
109	*Schisandra chinensis*	MF096278
110	*Schisandra chinensis*	KX815921
111	*Schisandra chinensis*	KX815922
112	*Schisandra chinensis*	KX815923
113	*Schisandra chinensis*	KX815924
114	*Schisandra chinensis*	KX815925
115	*Schisandra chinensis*	KX815926
116	*Schisandra chinensis*	KX815927

117	*Schisandra chinensis*	KX674907
118	*Schisandra chinensis*	KX674856
119	*Schisandra chinensis*	KX674855
120	*Schisandra chinensis*	KT285119
121	*Schisandra chinensis*	KT285120
122	*Schisandra chinensis*	KT285121
123	*Schisandra chinensis*	KP689675
124	*Schisandra chinensis*	KP689676
125	*Schisandra chinensis*	KP689677
126	*Schisandra chinensis*	KP689678
127	*Schisandra chinensis*	KP689679
128	*Schisandra chinensis*	KT898226
129	*Schisandra chinensis*	AF263441
130	*Schisandra sphenanthera*	KP893168
131	*Schisandra sphenanthera*	KP893169
132	*Schisandra sphenanthera*	KP893170
133	*Schisandra sphenanthera*	KP893171
134	*Schisandra sphenanthera*	KP893172
135	*Schisandra sphenanthera*	KP893173
136	*Schisandra sphenanthera*	AF163705
137	*Schisandra sphenanthera*	KY523609
138	*Schisandra sphenanthera*	KP689644
139	*Schisandra sphenanthera*	KP689645
140	*Schisandra sphenanthera*	KP689646
141	*Schisandra sphenanthera*	KP689647
142	*Schisandra sphenanthera*	KP689648
143	*Schisandra sphenanthera*	AF263437

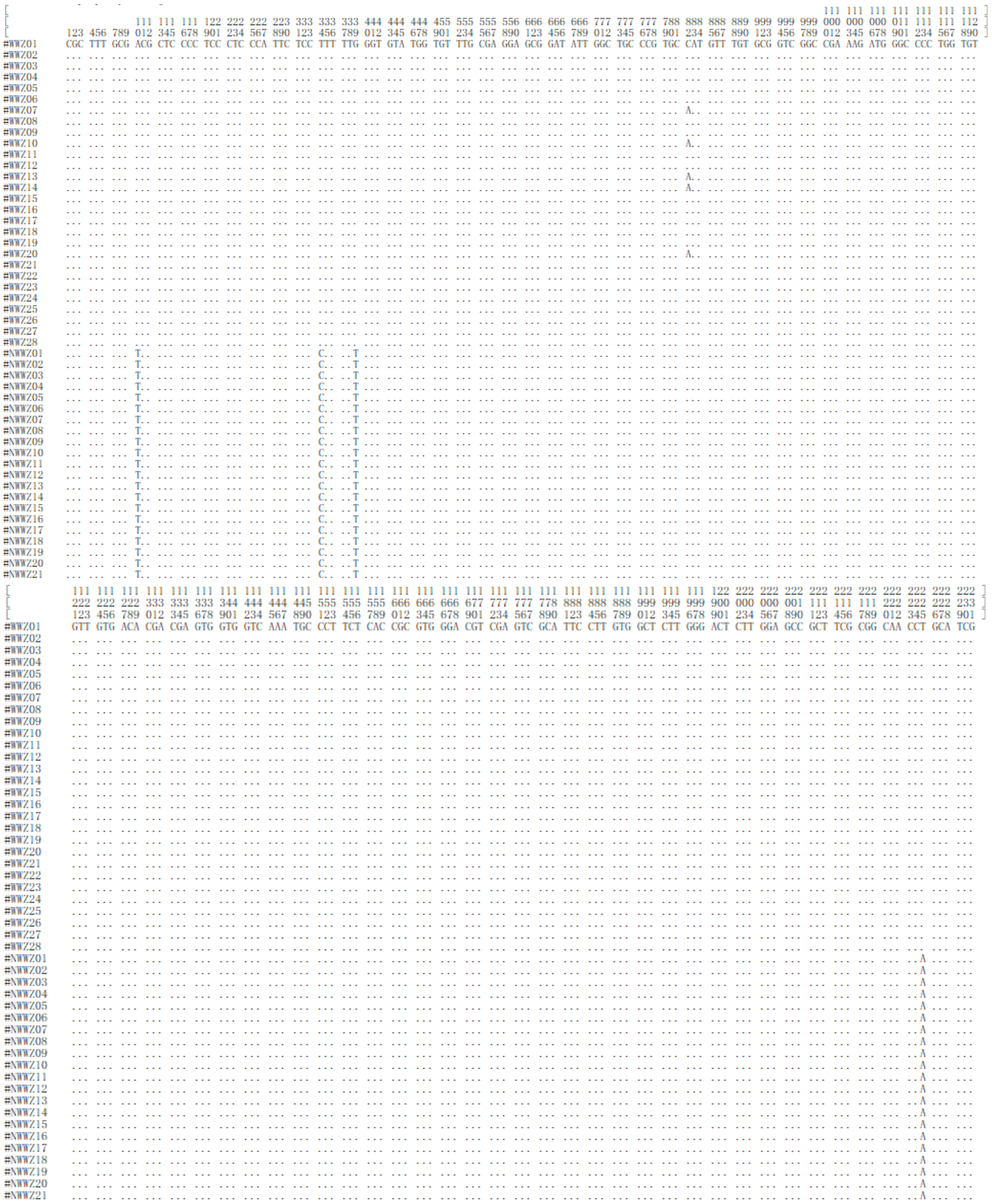

Figure S1. Alignment of the ITS2 region of experimental materials.
